# The Determinants of Long-Term Outcomes After Colorectal Cancer Surgery: A Literature Review

**DOI:** 10.7759/cureus.74985

**Published:** 2024-12-02

**Authors:** Olorungbami K Anifalaje, Charles Ojo, Oluwaseyi T Balogun, Fikayo A Ayodele, Abeeb Azeez, Shirley Gabriels

**Affiliations:** 1 Surgery, Mid Yorkshire NHS Foundation Trust, England, GBR; 2 General Surgery, Craigavon Area Hospital, Southern Health and Care Trust, Craigavon, GBR; 3 Dietetics, NHS Dumfries and Galloway, Dumfries, GBR; 4 Internal Medicine, Euracare Multi-Specialist Hospital, Lagos, NGA; 5 Surgery, University Hospital Southampton NHS Foundation Trust, England, GBR; 6 Emergency Department, Sandwell and West Birmingham NHS Trust, Birmingham, GBR

**Keywords:** colorectal cancer surgery, long term outcomes, post-operative outcomes, quality of life, recurrence rates

## Abstract

Colorectal cancer (CRC) is a common malignancy associated with high mortality. Surgical care is an effective colorectal cancer management technique, and it is therefore crucial that a review of the determinants of patients' long-term outcomes after CRC surgery is conducted. This article aims to provide healthcare professionals and policymakers with insights into the determinants of long-term outcomes following CRC surgery while acknowledging the interconnected impact of the early recovery and post-operative periods. For this review, PubMed and Google Scholar were used to search for literature on the determinants of long-term outcomes of patients post-colorectal cancer surgery. The determinants included pre-operative factors, CRC surgery factors (anatomical location of the lesion, select operative techniques, and cancer disease stage), adherence to the Enhanced Recovery After Surgery (ERAS) guidelines, post-operative complications, presence of an ostomy, physical activity levels, psychosocial factors, recurrence, and follow-up strategies. Selection criteria were published articles between 1994 and 2024 on colorectal cancer, its surgery, and determinants of outcomes. Several key determinants influence long-term outcomes following colorectal cancer surgery, including preoperative factors, CRC surgery factors, adherence to the ERAS guidelines, postoperative complications, the presence of an ostomy, physical activity levels, psychosocial factors, recurrence, and follow-up strategies. These determinants collectively impact survival, quality of life, functional recovery, and psychosocial well-being. On the one hand, negative outcomes following colorectal cancer surgery are often linked to preoperative factors such as poor nutritional status, sarcopenia, and inadequate adherence to ERAS guidelines during the perioperative period. Minimally invasive surgeries, while as effective as open surgeries for early-stage CRC, may be less suitable for advanced stages and often involve prolonged operating times - a factor linked to poorer outcomes. Complications of CRC surgery, such as anastomotic leakage, chronic surgical site pain, bowel dysfunction, and urological issues, further contribute to negative long-term outcomes. High recurrence rates are also linked to poor prognoses, emphasizing the importance of regular surveillance and timely interventions, though these can lead to patient anxiety and overtreatment. The presence of an ostomy can impact psychosocial adjustment and overall quality of life, further influencing long-term outcomes. On the other hand, positive outcomes are associated with regular physical activity post-surgery, which significantly aids long-term recovery irrespective of preoperative activity levels. Psychosocial support networks also play a crucial role in mitigating mental health challenges often faced after CRC surgery. Collectively, these determinants underscore the complexity of long-term outcomes in colorectal cancer surgery and highlight the importance of a holistic approach to patient care.

## Introduction and background

Colorectal cancer (CRC) is a global health concern, ranking as the second most fatal cancer of the 21st century. As of 2020, an estimated 1.9 million new cases and 0.9 million deaths were reported worldwide [1], with developed countries having the most significant CRC incidence and mortality [2]. In developing countries, a rise in colorectal diagnosis over the years has been noted, and it is believed that these figures may even be underestimated due to the evolving nature of healthcare data management systems in such regions [3].

Surgery is the recommended treatment option for colorectal cancer and is the case in about 75% of cases; however, with advanced disease conditions or compelling surgical contraindications, neo-adjuvant care may be a suitable alternative, accounting for about 25% of colorectal malignancies [4].

Over the years, research has rightly focused on evaluating survival and complications post-colorectal cancer surgery, further underscoring its significance [5-8]. These frequently studied outcomes after surgery are largely multifactorial. While they vary with the type of surgery, pre-operative factors, post-operative complications, the presence of an ostomy, physical activity levels, psychosocial factors, recurrence rates, and follow-up strategies, the results of these determinants and their long-term outcomes have often been contrasting - thus necessitating this review [9-13]. These determinants can influence long-term outcomes after CRC surgery, encompassing survival, quality of life, functional recovery, and psychosocial well-being. Reviewing these multidimensional factors is crucial, as they provide insights into parameters that can guide interventions and optimize patient-centred care [5,14-16].

Methodology

For this review, PubMed and Google Scholar were used to access literature highlighting the determinants of outcomes following colorectal cancer surgery while acknowledging the interconnected impact of the early recovery phase and the post-operative period. This paper was written with a thematic approach highlighting these determinants and how they affect outcomes. The research strategy involved (Outcomes OR Outlook OR Effect OR Recovery) AND (Colorectal OR Colon OR Rectal OR Abdominal) AND (Surgery OR Resection) AND (Cancer OR Malignancy OR Tumour OR Oncology). The inclusion criteria were human studies centred on colorectal cancer care surgery, written in English and published between 1994 and 2024. Exclusion criteria included animal studies and papers not written in the English language.

## Review

Pre-operative factors

Pre-operative factors influencing long-term outcomes after colorectal cancer surgery include nutritional status, sarcopenia, and bowel preparation, amongst others.

Nutritional status plays a particularly significant role, with poorer outcomes observed in patients at nutritional risk, moderately malnourished, or reduced visceral adipose tissue (VAT). Optimizing nutrition pre-operatively has been shown to significantly lower mortality rates, even in older patients, those with high-risk genetic mutations, and metastatic colorectal cancer cases [17-19]. Notably, pre-operative nutritional rehabilitation has improved outcomes, including a return to pre-surgical function and reduced mortality [20,21]

Sarcopenia, characterized by low muscle quality or quantity, is associated with poor outcomes following colorectal cancer surgery. These include longer hospital stays, a higher risk of post-operative sepsis, and adverse survival metrics such as disease-free survival, recurrence-free survival, cancer-specific survival, and overall survival. Best practices to address sarcopenia include nutritional support with high-energy and protein supplements, anabolic-orexigenic agents (ghrelin, anamorelin), and exercise programs, all of which aid in preserving muscle mass and improving outcomes [22,23].

Globally, there does not appear to be a consensus for the use of mechanical bowel preparation and oral antibiotic prophylaxis prior to colorectal cancer surgery. The UK’s regulatory body, the National Institute for Health and Care Excellence (NICE), in its updated 2019 guidelines, appears to shy away from recommending their routine use [24]. However, more recent studies advocate for their use, citing reduced rates of anastomotic dehiscence, surgical site infections, and overall morbidity [25-27]. Similarly, in its 2018 guidelines, the WHO recommends the routine use of mechanical bowel preparation and oral antibiotic prophylaxis to lower the risk of surgical site infections and other morbidities [28].

Surgical factors

Colorectal cancer resection entails removing the tumour's principal vascular pedicle and lymphatics, creating an anastomotic margin free of cancer cells, and resecting any organs or structures connected to the tumour [29]. Favourable colorectal cancer anatomical locations, selected operative techniques, and non-advanced disease stages, among other factors, are associated with improved survival outcomes after surgery. Conversely, poor survival outcomes, including higher mortality rates following surgical resections, are higher in patients with unfavourable tumour locations and advanced disease stages [30,31].

The anatomical location of colorectal cancer affects prognosis post-operatively. Survival outcomes are best in patients undergoing sigmoid colectomies, moderate at best, and similar in resections for independent caecal cancer, ascending hepatic flexure, and transverse colon cancer, and worse in cases involving abdominoperineal excision of the rectum (APER) for rectal malignancies [32].

Laparoscopic and open colorectal cancer surgeries demonstrate similar rates of cure, recurrence, survival outcomes, and health-related quality of life (HRQoL) [33-36]. However, laparoscopic surgeries are often less preferred for advanced metastasis due to limited access to multi-organ resections [37]. Despite this, laparoscopic procedures offer better cosmetic outcomes [38] and typically involve intracorporeal anastomosis, which, while taking longer to construct compared to the extracorporeal anastomosis used in open surgeries, has been shown to reduce post-operative complications, shorten hospital stays, and lower reoperation risks [39,40]. Technical challenges, particularly for less experienced surgeons, can further prolong operating times during laparoscopic CRC surgeries [41] - a factor consistently associated with poorer long-term outcomes. Extended operating times can increase the risks of surgical trauma, prolonged exposure to airborne pathogens, venous thromboembolism, immune suppression, nutrient malabsorption, and surgical team fatigue, all of which contribute to poorer outcomes [42].

Complete excision, which meticulously dissects along the anatomical planes and ensures high ligation of the appropriate vasculature, is associated with improved survival outcomes; this is usually the case for CRC stage I-III [43-45]. However, CRC stage IV resections have a poor prognosis, and this is most pronounced in patients with significant lymphatic spread, hepatic tumour load greater than 50%, microscopic and/or macroscopic residual tumour, and a lack of adjuvant care [46].

En-bloc excision of the tumour's lymphatic drainage together with the associated lymph nodes significantly affects the long-term prognosis following CRC surgery. With this surgical technique, many studies have unequivocally demonstrated that proper cancer staging is correlated with pathologists examining a more significant number of lymph nodes [47-49]. The total number of lymph nodes examined reflects how well the pathologist processes the specimen, enabling categorization based on the likelihood that the malignancy may reoccur or metastasize. A lower ratio of lymph nodes with metastasis to lymph nodes without metastasis has been linked to improved colorectal cancer survival [50,51].

ERAS guidelines

The Enhanced Recovery After Surgery (ERAS) guidelines, established over many years, largely capture globally accepted recommendations for colorectal cancer surgery. They provide comprehensive pre-operative, intraoperative, and post-operative care guidance to reduce complications and shorten hospital stays [52]. However, implementation varies across healthcare institutions due to local policy adaptations [53,54] and logistical or financial constraints limiting full adherence [55,56]. Despite these challenges, the guidelines have directly translated into significant cost savings for healthcare systems [57] and improved patient satisfaction post-surgery [58].

In CRC surgery, the ERAS guidelines emphasize pre-operative patient education on the surgical process, their role in recovery, and setting realistic expectations alongside nutritional supplementation, pain management, antibiotic prophylaxis, and venous thromboembolism prophylaxis to enhance recovery and reduce complications. In the intra-operative period, an emphasis is placed on preserving core body temperature and ensuring judicious use of fluid therapy. Post-operatively, the guidelines collectively stress balanced fluid regimens, early gut stimulation, careful drain management, and early mobilization to improve outcomes [53].

Post-operative complications

Surgical complications affecting patients' long-term outcomes after CRC surgery include anastomotic leakage, chronic surgical site pain, and bowel and urological problems, among others [59,60].

Anastomotic leakage is a particularly concerning complication of CRC surgery, as it is linked to significant adverse outcomes such as an increased risk of sepsis, wound dehiscence, wound infection, post-operative hemorrhage, abdominopelvic collections, medical complications, and the need for further surgeries. Despite advancements in surgical techniques, anastomotic leakage remains relatively common, occurring in up to 10% of patients after CRC surgery. It is more frequently observed in left-sided CRC surgeries, emergency procedures, open surgeries, multi-organ resections, and rectal surgeries, with a shorter tumour distance to the anal verge further increasing this risk. Anastomotic leakage is highly debilitating, leading to poor outcomes such as reduced overall survival, disease-free survival, and cancer-specific survival, along with higher rates of both local and distant cancer recurrence [59].

Chronic surgical site pain - ongoing pain for more than 12 weeks - is an established complication of CRC surgery and occurs in about 12% of patients after surgery [61]. Chronic pain frequently interferes with activities of daily living and recovery. Predictors of developing chronic pain after CRC surgery include young age at surgery, pre-existing mental health disorders, pre-existing somatic pain, longer length of surgery, high pain intensity on movement within 24 hours following surgery [62], more complex and invasive colorectal surgeries [63], and rectal cancer surgery with anastomotic margins less than 2 cm from the anatomically significant anal verge [64].

During CRC surgery, injuries to the nerves controlling the internal anal sphincter (IAS) and the external anal sphincter (EAS) can occur, particularly in the case of an inter-sphincteric resection for malignancy significantly impacting continence [65]. For instance, a study involving 961 patients who underwent low anterior resection (LAR) surgery for rectal cancer identified common issues, including common complaints of flatus and stool, bowel frequency, clustering, and urgency. These complications varied depending on factors such as partial or total mesorectal excision (TME), associated radiotherapy, and a tumour height of 5 cm or more [60,66]. Interestingly, up to 72% of patients after LAR surgery report bowel dysfunction [67].

CRC surgeries, including LAR, TMEs, and abdominoperineal resection of the rectum (APER) surgeries, have also been associated with an increased risk of developing urological problems post-operatively, with complication rates of up to 50% [68] and independent risk factors being a previous urological history and co-existing diagnosis of diabetes mellitus [69]. However, these deficits are usually temporary and often resolve within 12 months post-surgery and may include a reduction in void volume and incomplete bladder emptying [70]. The anatomical basis of the high incidence of urinary dysfunction is due to the close relationship of the autonomic nervous plexus to the endopelvic fascia, and a disruption in these nervous plexus gives rise to the myriad of problems previously highlighted [71].

Ostomy as a determinant of long-term outcomes

Ostomies, which can either be temporary or definitive, have been found to have significant positive and negative effects on patients after CRC surgery [72-75]. Temporary stomas are more likely to be constructed in patients to protect a distal anastomosis and with a plan of revision surgery. In contrast, definitive stomas are more commonly done in advanced malignancies. Most often, a stoma is constructed in CRC patients with left-sided colonic tumours, rectal malignancies, metastatic disease, non-radical procedures, advancing age groups, and open colorectal surgeries [76,77].

**Figure 1:**
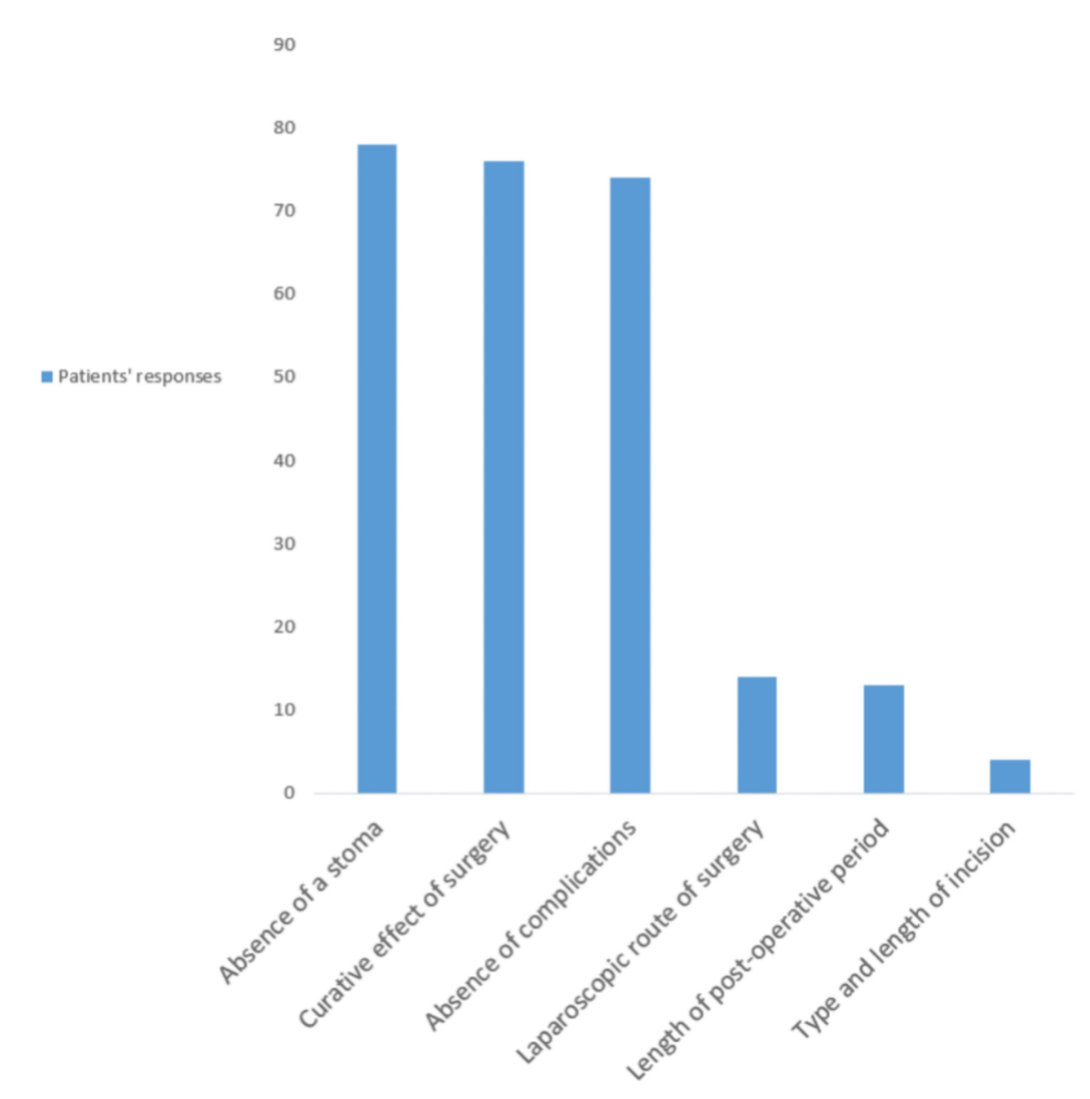
Patient's perceived predictors of optimal quality of life after colorectal cancer surgery Original work of authors. A study highlighting the importance of avoiding a stoma to patients as the most important factor after CRC surgery over being cured of primary pathology, post-operative complications, and other surgical factors.

Stomas constructed as the first surgical intervention to relieve an imminent obstruction or the debilitating consequences of an anastomotic leak following CRC surgery are associated with the most advantageous outcomes [78-81]. However, they have been shown to cause significant distress, so much so that patients in a cross-sectional study of 167 respondents of a defined retrospective population after CRC surgery mentioned that their quality of life improved by not having a stoma (78%), being cured of the primary pathology (76%), and avoiding complications (74%), the use of laparoscopy for surgery (14%), length of hospital stay post-operatively (13%), and incision type and length (4%) [30,71,81-84]. Figure 1 depicts this through a bar chart.

An ostomy is also associated with an increased risk of readmission after discharge and reduced quality of life. In a study involving 10,882 post-operative patients, 11.4% of them experienced 30-day readmission following colorectal surgery. A further 11.9% were readmitted within the next 31 to 90 days, giving a total readmission rate of 23.3% within 90 days. In the first 30 and 90 days, 1.4% and 5.2% of patients experienced two or more readmissions, respectively. The presence of an ostomy was a significant reason for readmission [85].

The type of stoma constructed affects patients' long-term outcomes post-CRC surgery. Loop transverse colostomies are associated with poor recovery due to an increased risk of stoma sepsis, prolapse, and ileus. While loop ileostomies, due to being more proximally sited, result in high output and an increased risk of dehydration, they may be linked to quicker post-operative recovery as they have a reduced risk of stoma sepsis, prolapse, and ileus [86]. Furthermore, stoma complications, including parastomal hernias, occur in up to 30% of patients post-operatively and are a common cause of reoperation, with a further need for surgery found in up to 12.5% of patients after initial surgery and, expectedly, poorer outcomes [87,88].

Fortunately, the side effects that the presence of a stoma causes can be shortened since many studies nowadays have shown that stomas can now be reversed within eight to thirteen days after primary surgery, as opposed to the traditional three-month window [84,88-90]. However, studies have also shown that up to 35% of temporary stomas will never be reversed [91,92], with independent predictors of non-reversal of intended temporary stomas, including anaemia, impaired renal function, and metastatic disease at the peri-operative period for reversal surgery [93].

Physical activity

In the absence of complications, most patients with colon cancer recover to normal function after surgery within six weeks. However, individuals with rectal cancer surgery may need up to eight weeks to fully recover [71]. Mobilization after surgery is often recommended to reduce the risk of adverse events and aid recovery [94]. Following CRC surgery, a rise in moderate-to-vigorous physical activity is linked to a faster recovery of physical functioning, and this improvement is demonstrable irrespective of pre-operative physical activity levels in reasonably well patients [95]. For instance, a prospective cohort study done that used accelerometers to measure the quantity of aerobic exercise performed and recorded in steps throughout the peri-operative period of colorectal cancer patients showed that there was a significant correlation between the number of post-operative footsteps and reduced risk of disability, loss of independence, fatigue, and insomnia [96]. These findings support the argument that effective post-operative rehabilitation is a significant parameter that enhances positive outcomes [96,97]. In fact, they also reduce the chance of developing chronic illnesses like diabetes and heart diseases and the risk of tumour recurrence [98,99]. They improve the quality of life, functional status, strength, and tolerance of surgical side effects and reduce the risk of complications, including lymphoedema and asthenia, after CRC surgery [100-103].

Even though physical activity has been linked to improved patient outcomes, patients post-CRC surgery find it difficult to adhere to recommended physical activity regimens. For instance, in a study done to evaluate levels of physical activity after CRC surgery, only 23.5% of CRC survivors adhered to recommended exercise regimens [104]; this low figure was due to post-operative complications, including having an ostomy, poor bladder control, psychological factors, a perceived lack of social support, and limited awareness of the benefits of exercise in improving outcomes after surgery [105].

Psychosocial factors

**Figure 2:**
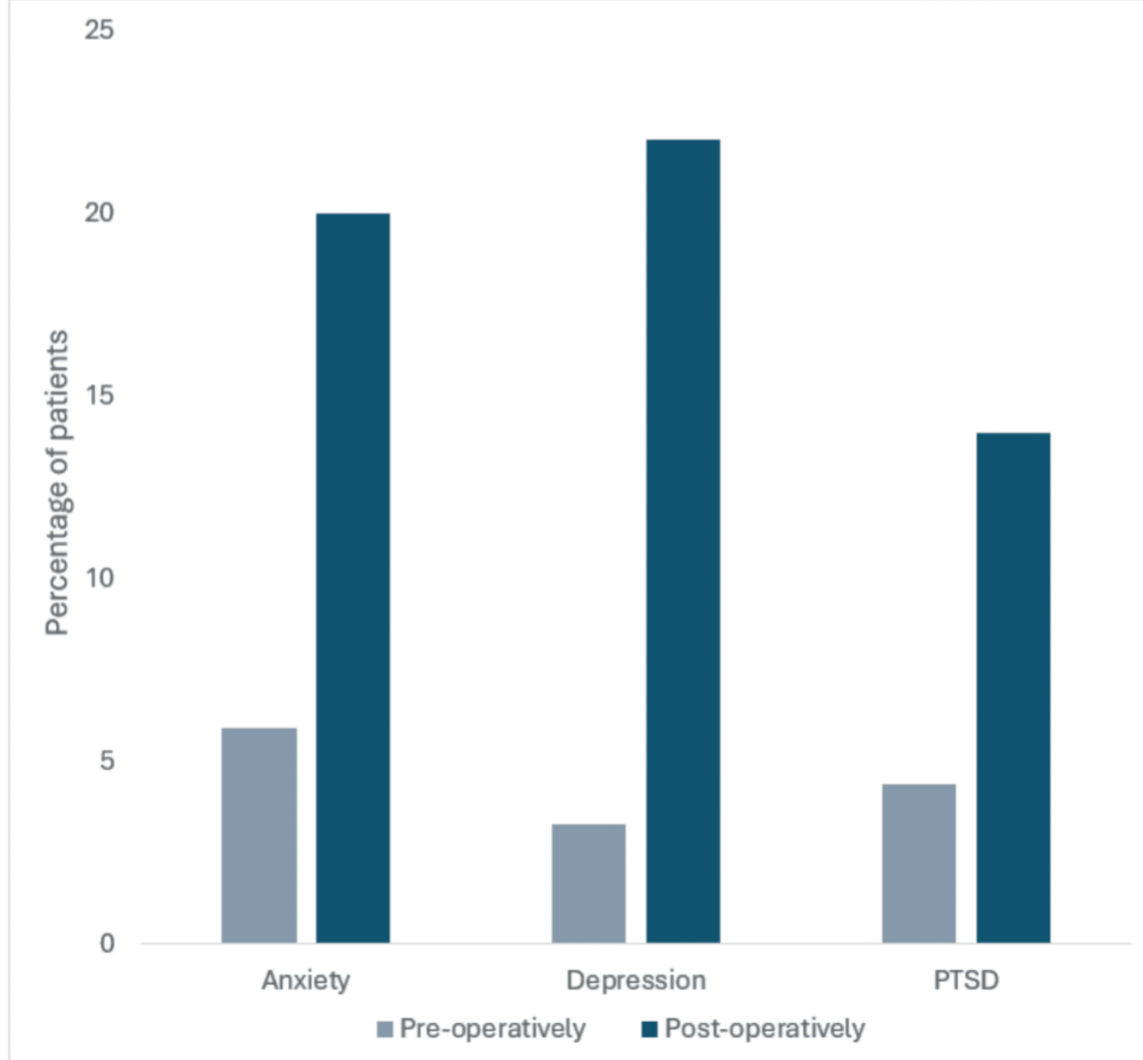
Anxiety, depression, and post-traumatic stress disorder rise steeply after colorectal cancer surgery Original work of authors. The level of Anxiety, depression, and PTSD in the average population is 5.9%, 3.3%, and 4.4%, respectively. These levels rise to 20%, 22%, and 14%, respectively after CRC surgery.

Following colorectal cancer surgery, higher levels of support have been linked to improved health outcomes and HRQoL [106,107]. Psychosocial factors include patients' baseline mental health state, support from partners, family members, close friends, and healthcare practitioners [108-110]. Studies have shown that while the levels of anxiety, depression, and post-traumatic stress disorder (PTSD) are estimated to affect 5.9%, 3.3%, and 4.4% of the general population, respectively, following colorectal surgery, this number dramatically increases, with the prevalence of anxiety, depression, and PTSD ranked at 20%, 22%, and 14%, respectively [111]. This is represented in Figure 2. A diagnosis of depression after surgery is particularly problematic as they have been shown to have worse in-hospital and long-term outcomes [112]. Therefore, an early and continued assessment of support would allow for focused interventions to enhance recovery [108]. 

In a cohort study evaluating the influence of social support (the presence of support at home - including partners, family members, and close friends) on patients after curative colorectal cancer surgery, levels of social support declined by about 29% from baseline after primary CRC surgery, resulting in poor overall physical and mental health [107].

With CRC surgery being a psychosocially challenging venture, surgeons who are frequently the first medical professionals to discuss a new colorectal cancer diagnosis and treatment options with patients in-depth have a crucial role to play as they first-hand can experience and rationalize how patients handle the anxiety associated with receiving a diagnosis and following surgery [109]. Healthcare professionals are therefore encouraged to screen patients for distress, recognize and enhance patients' coping mechanisms, enable a solid social support system, and give patients the choice to seek additional help [109].

As improved recovery is associated with good pre-operative mental states and overall life satisfaction, patient may be better equipped to deal with the impact of colorectal surgery and recuperate faster, an important determinant of successful holistic care and an important outcome after CRC surgery [113].

Recurrence of colorectal cancer

Although, following CRC surgery, up to one in three patients will experience a recurrence - with the majority resulting in death - the prognosis is improved through effective follow-up strategies and screening [13]. While the optimal follow-up period after CRC surgery is debatable, recurrence is more prevalent in the first two to three years following surgery, and it either occurs as locoregional or distant metastasis, commonly involving the lungs or the liver [114]. CRC recurrence may be symptomatic or asymptomatic, but post-operative screening methods, including tumour marker testing, radiological imaging, and endoscopy, can accurately detect recurrence [115].

The symptomatology of CRC recurrence after initial surgery plays a role in patients' long-term outcomes. For instance, in a study that followed up patients five years post CRC surgery, symptomatic patients who usually present with new symptoms, including new abdominal pain, change in bowel habits, and weight loss, are less likely to be offered further surgeries to manage recurrence and thus have poorer outcomes, and this is further supported by the fact that symptomatic patients tend to have multisite recurrence, further complicating survival outcomes [115].

Locoregional recurrence post-initial CRC surgery also plays a role in patients' long-term outcomes. This involves recurrence at the site of initial anastomosis, adjoining mesentery, and peritoneum. It may occur in up to 11.5% of patients post-CRC surgery, and if, at primary surgery, an advanced disease was found, locoregional recurrence rates are even higher [116,117]. Locoregional recurrence causes survival rates to be about nine months; however, if further surgeries are performed toward complete tumour clearance, survival may improve over five years [117,118]. Low locoregional recurrence rates may occur without adjacent organs or significant lymphatic involvement, which portrays better long-term survival outcomes [116].

Distant metastasis post-CRC surgery causes distressing symptoms and reduced survival rates. Whilst hepatic metastasis results in symptoms including fatigue, weight loss, and ascites [30,119,120], pulmonary metastasis causes difficulty with breathing and generalized fatigue, all of which worsen over time until they significantly impair activities of daily living [121,122]. Furthermore, research has shown that liver metastasis - which occurs in up to 10% of patients post-CRC surgery [123] - is much more extensive in right-sided colorectal cancer and has a resultant higher mortality risk when compared to liver metastasis from left-sided colorectal cancer [124]. Survival rates in hepatic recurrence of CRC are about 29 months without surgery, and even with surgery, there is only a marginal improvement in survival rates, with only 36% of patients surviving for up to five years - this is mainly due to multiple metastatic deposits, large tumour diameter, difficulty with achieving a clear resection margin, and a need for further surgery [125-127]. In the same vein, survival rates for pulmonary metastasis following colorectal cancer surgery, which may occur in up to 3.5% of patients [128], have survival rates of less than a year, and again it only marginally improves to five years in just about 22.2% of patients - which is especially due to the presence of hilar lymph node metastasis [129-131].

Follow-up strategies

Follow-up strategies after CRC surgery have positive and negative effects. Although standardized to involve serological, radiological, and endoscopic investigations, these strategies may vary with disease stage, patient fitness, and life expectancy, especially within the first five postoperative years - where 95% of CRC reoccurs [114,132]. For instance, while the NICE UK recommends scheduled bi-annual serum carcinoembryonic antigen (CEA) testing, yearly computed tomography scans (CT scans) of the thorax and abdomen, and colonoscopy done at the first and third year after primary surgery done with curative intent [133], stage I CRC has significantly lower reoccurrence rates of less than 10% [134] as opposed to more advanced CRC stages, which may reoccur in about 40% of patients after initial surgery and may need more intensive follow-up [135,136].

While scheduled follow-up assessment for patients after CRC surgery has its benefits in offering reassurance to patients that recurrence is likely to be detected much more quickly, it has also been shown to be a source of stress for patients, resulting in reduced quality of life, thus necessitating a balance of risk to benefit [136]. Although regular follow-up is beneficial, as these clinical interactions enable healthcare professionals to pick up on long-term complications of CRC surgery and associated patients' comorbidities in a bid to improve outcomes, frequent assessments and investigations increase the risk of overtreatment and unnecessary interventions in patients who may not necessarily benefit from it due to significant comorbidities or limited life expectancy - this reinforces the importance of individualized risk assessment of patients profiling prior to intervention [135,136].

Worthy of note is the role of surveillance colonoscopies/proctosigmoidoscopies and CEA testing following CRC surgery. Although surveillance colonoscopies/proctosigmoidoscopies are invasive and relatively expensive, they can detect the recurrence of CRC at anastomotic sites early and may be particularly useful, especially in patients with sporadic colorectal cancer, which are known to have a threefold risk of recurrence [137,138]. On the other hand, serum CEA testing offers a less invasive and inexpensive means to track CRC recurrence. Several studies have shown that a CEA level of >5 ng/ml positively predicts recurrent disease in about 80% of cases and should prompt a more extensive workup before further surgical interventions [139-141]. Furthermore, when combined, colonoscopy and CEA appear to have the most significant impact on predicting recurrence, which helps guide surgical management towards improving patients outcomes [135,136].

## Conclusions

A complex interplay of pre-operative factors, surgical factors, adherence to ERAS guidelines, post-operative complications, physical activity levels, psychosocial support, the presence of an ostomy, recurrence rates, and follow-up strategies influences the long-term outcomes of patients following colorectal cancer surgery. Whilst optimal operating conditions, techniques, and reasonable follow-up strategies enhance recovery and reduce recurrence rates, pre-operative diagnosis of sarcopenia and malnutrition, poor adherence to ERAS guidelines, long-term ostomies, post-operative complications, lack of psychosocial support, and limited physical activity are associated with poor long-term outcomes. Ultimately, an integrated approach that addresses these factors will improve the likelihood of better patient outcomes. More studies focusing on refining these determinants to develop targeted interventions are needed to support patients through their recovery and enhance long-term outcomes.
